# Get Health‘e’: A Pilot Test of a Digital Health Literacy Intervention for Young Adults

**DOI:** 10.3928/24748307-20240723-01

**Published:** 2024-10

**Authors:** Jennifer A. Manganello, Kimberly F. Colvin, Molly Hadley, Kelsey O'Brien

## Abstract

**Background:**

Young adults face a number of health concerns and are typically beginning to manage their health on their own. At the same time, this age group is more connected to digital technology than ever before, and studies show that young adults can struggle with digital health literacy skills.

**Objective:**

The online Get Health‘e’ Program consists of six modules addressing digital health literacy skills, including topics such as internet searches and social media. This study evaluates the program based on a usability and pilot study.

**Methods:**

Recruited participants ranged in age from 18 to 24 years and were predominantly White women. After an eligibility screening, half of the participants were randomly assigned to the program group. All 131 participants were administered two surveys, 2 weeks apart; the program group had access to the program for that week. Survey questions included demographics, the eHealth Literacy Scale (eHEALS), and six sets of five multiple-choice questions assessing digital health knowledge.

**Key Results:**

Both groups showed an improvement of 1.0 points on the eHEALS scale, but a repeated measures analysis comparing scores for the program and control groups indicated no significant difference in score improvement between the two groups, *F*(1, 129) = 0.003, *p* = .96. The program group had an average improvement in quiz scores (from pre to post) from 1.6 to 2.3 which was better than score improvement in the control group across the six modules. The majority of participants in the program group found the program to be useful (67.2%) and informative (59.4%), and 93.8% said they would recommend it to someone they know.

**Conclusions:**

The Get Health‘e’ program was well received by participants and led to an increase in digital health knowledge. Programs like this have the potential to raise awareness among youth about how to access and evaluate health information online. [***HLRP: Health Literacy Research and Practice*. 2024;8(4):e224–e235.**]

## Plain Language Summary

Although young adults are more connected to digital technology than ever before, studies show they still struggle with digital health literacy skills. The online Get Health‘e’ Program consists of six modules addressing digital health literacy skills, including topics such as internet searches and social media. The program was well received by participants and increased digital health literacy knowledge.

Young adults face many health concerns and are experiencing health declines, particularly since the coronavirus disease 2019 (COVID-19 pandemic). Although they are typically beginning to manage their health on their own, studies show that young adults can struggle with digital health literacy skills (Dadaczynski et al., 2021; Frings et al., 2022; Patil et al., 2021; Taba et al., 2022). At the same time, this age group is more connected to digital technology than ever before. The Get Health‘e’ Program was designed to help build digital health literacy skills for young adults by addressing topics such as internet searches and social media.

Young adulthood (age 18 to 24 years) is a formative time for developing healthy habits and improving future health outcomes, but this age group has demonstrated declines in multiple aspects of health and poor health-related behaviors (Bonnie et al., 2015). The prevalence of obesity and mental health challenges continue to steadily increase, and many young adults engage in unhealthy activities that include substance use (M. J. Park et al., 2014; Substance Abuse and Mental Health Services Administration, 2022) and poor nutrition (Jahangard et al., 2019). Just over half of young adults have at least one chronic condition (Watson, 2022). Young adults from vulnerable populations have risk factors for health disparities (Wickrama et al., 2022). For instance, race and ethnicity have been found to impact health behaviors and outcomes in this age group (Bonnie et al., 2015; Monroe et al., 2023). In addition, young adults have historically been more likely than any other age group to be uninsured (Mulye et al., 2009). While the Affordable Care Act has helped (Courtemanche et al., 2019), there are still gaps in coverage. Data from 2019 shows that adults ages 19 to 34 years had an uninsurance rate of 15.6%, the highest of any age group (Conway, 2020). Given the various health and health care issues facing young adults, interventions are needed to ensure that this important population can make informed choices and get needed services to manage their health.

The period between late adolescence and early adulthood is a critical point in development since this is when individuals are learning to accept responsibility for oneself, become financially independent, and make independent decisions (Arnett, 2007); they are also beginning to interact with the health system on their own. Given this, it is critical for young adults to develop strong health literacy skills. Health literacy (HL) is defined as “the degree to which individuals can obtain, process, understand, and communicate about health-related information needed to make informed health decisions” (Berkman et al., 2010) (p.16). Health literacy plays a significant role in one's ability to understand health information and navigate the health system, and incompetency in this area has been linked with a number of poor health outcomes (Berkman et al., 2011). Sansom-Daly et al. (2016) completed a systematic review to assess health literacy in adolescents and young adults and concluded that 60% displayed adequate levels. Additionally, poorer health literacy was associated with some negative health outcomes. Another study confirmed health literacy is associated with health behaviors in this age group (Jahangard et al., 2019). While much information exists for health literacy and adults, the gaps in studies that focus on health literacy for adolescents and young adults identified several years ago (Manganello, 2008) remain, suggesting that more work is needed (Bröder et al., 2020).

At the same time, this group is more connected to digital technology than ever before. A Pew survey from 2021 found that 98% of adults age 18 to 29 years use the internet, and 94% have a smartphone (Fox, n.d.). Therefore, not only is general health literacy important, but young adults must have sufficient literacy within the context of eHealth, or digital health literacy (Kim & Xie, 2017). Digital health literacy is defined as “the ability to seek, find, understand, and appraise health information from electronic sources and apply the knowledge gained to addressing or solving a health problem” (All of Us Research Program, n.d.). It is often used interchangeably with the term eHealth literacy, which is the ability to assess health information from electronic sources and apply the knowledge gained to addressing a health-related concern (Smith & Magnani, 2019). A systematic review concluded that many young adults lack eHealth literacy skills, especially as it relates to searching for, retrieving, using, and evaluating health information found online (Stellefson et al., 2011). Another review found that adolescents vary in their abilities to search for and assess online health information and are in need of resources to develop these skills (Freeman et al., 2018).

Although digital health literacy is now seen as a social determinant of health, there are few interventions available to build digital health literacy skills (AriasLópez et al., 2023). Implementing interventions online is ideal for this population given their ubiquitous technology use. Many successful online interventions have been developed for young adults, including college students, with respect to health education around topics such as nutrition and physical activity (Belogianni et al., 2023; Nikolaou et al., 2015), mental health (Kirschner et al., 2022), sexual health (Bull et al., 2012), diabetes (An et al., 2023), and more (Heckman et al., 2015; Oguntoye et al., 2022). However, we identified only one that included general digital health literacy skill building (Roh & Won, 2023). This study included female college students in Korea and assessed an online intervention designed to improve knowledge about different health topics such as sleep and nutrition, as well as how to search for health information online. This study found the intervention succeeded in improving eHealth literacy skills as measured by the eHEALS (Roh & Won, 2023). However, this study did not include males and did not address other aspects of digital health such as how to use wearable devices and patient portals.

Research with other age groups also suggests that online digital health literacy interventions can be successful. An online course for school-aged children in Germany was found to be successful at increasing both health literacy and digital health literacy skills (König et al., 2022). Online massive open online courses for pregnant and lactating women (Álvarez-Pérez et al., 2022) and diabetes (Álvarez-Pérez et al., 2021) were also found to be successful in developing digital health literacy skills. A review of digital health literacy interventions for older adults found that most had an impact on eHealth literacy scores (Dong et al., 2023). Given that interventions for digital health literacy are limited, and reviews suggest almost none have focused on young adults, this study was designed to develop and test an intervention to enhance digital literacy skills for youth ages 18 to 24 years.

## Purpose

To address the need to build digital literacy skills for young adults, we developed the Get Health‘e’ online program and conducted a pilot evaluation. Participants in the pilot study were randomly assigned to either have access to the program or be in the wait-list control group, who received access after the study was concluded. Using data collected from the pilot study, we evaluated the efficacy of the program, as well as participants' feedback on their experience with the program.

## Method

### Get Health‘e’ Program

This program was informed by the Framework for Adolescent Health Literacy, which emphasizes the role of media use on health literacy (Manganello, 2008), and the Health Literacy Skills Framework, which states that an important component of health literacy is “information seeking and ehealth” (Squiers et al., 2012). Both models point to the role that health literacy plays in health outcomes.

We designed the program as an online course with plain language text, brief videos of 3 to 5 minutes, and colorful images and graphics. The six modules addressed: (1) an overview of digital health and digital health literacy, (2) the best ways to search for and evaluate health information online, (3) patient portals, (4) social media and health information, (5) health-related phone apps, and (6) wearable health devices such as Fitbits. After each module, there was a brief 5-question quiz to assess knowledge. Some of the content was newly created, while other content was pulled from websites and resources developed by government agencies, health organizations, and universities. Undergraduate and graduate students worked on developing the content and video scripts so that the information was generated by people in the target age group. **Table 1** provides a description of and sample question for each module. The study was approved by the University at Albany Institutional Review Board.

**Table 1:**
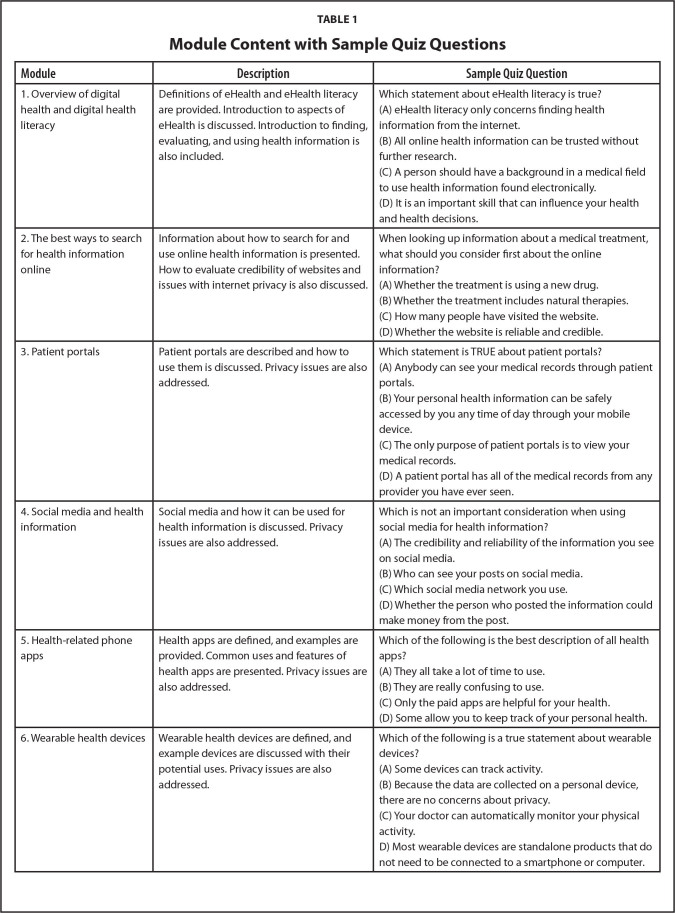
Module Content with Sample Quiz Questions

**Module**	**Description**	**Sample Quiz Question**
1. Overview of digital health and digital health literacy	Definitions of eHealth and eHealth literacy are provided. Introduction to aspects of eHealth is discussed. Introduction to finding, evaluating, and using health information is also included.	Which statement about eHealth literacy is true? (A) eHealth literacy only concerns finding health information from the internet. (B) All online health information can be trusted without further research. (C) A person should have a background in a medical field to use health information found electronically. (D) It is an important skill that can influence your health and health decisions.
2. The best ways to search for health information online	Information about how to search for and use online health information is presented. How to evaluate credibility of websites and issues with internet privacy is also discussed.	When looking up information about a medical treatment, what should you consider first about the online information? (A) Whether the treatment is using a new drug. (B) Whether the treatment includes natural therapies. (C) How many people have visited the website. (D) Whether the website is reliable and credible.
3. Patient portals	Patient portals are described and how to use them is discussed. Privacy issues are also addressed.	Which statement is TRUE about patient portals? (A) Anybody can see your medical records through patient portals. (B) Your personal health information can be safely accessed by you any time of day through your mobile device. (C) The only purpose of patient portals is to view your medical records. (D) A patient portal has all of the medical records from any provider you have ever seen.
4. Social media and health information	Social media and how it can be used for health information is discussed. Privacy issues are also addressed.	Which is not an important consideration when using social media for health information? (A) The credibility and reliability of the information you see on social media. (B) Who can see your posts on social media. (C) Which social media network you use. (D) Whether the person who posted the information could make money from the post.
5. Health-related phone apps	Health apps are defined, and examples are provided. Common uses and features of health apps are presented. Privacy issues are also addressed.	Which of the following is the best description of all health apps? (A) They all take a lot of time to use. (B) They are really confusing to use. (C) Only the paid apps are helpful for your health. (D) Some allow you to keep track of your personal health.
6. Wearable health devices	Wearable health devices are defined, and example devices are discussed with their potential uses. Privacy issues are also addressed.	Which of the following is a true statement about wearable devices? (A) Some devices can track activity. (B) Because the data are collected on a personal device, there are no concerns about privacy. (C) Your doctor can automatically monitor your physical activity. D) Most wearable devices are standalone products that do not need to be connected to a smartphone or computer.

### Usability Study

Before implementing the pilot program, we conducted a usability study (*n* = 10) to identify whether users would be able to navigate the system easily, would like the appearance of the system, and whether they had any feedback for changes. This phase of the project involved a brief online survey, exploration of the program, and semi-structured interview questions to solicit feedback. Participants were recruited through flyers and word-of mouth recruitment in the community.

Once a participant was recruited into the usability study, they were scheduled for a time to participate in a phone call session and were then provided with a link to the survey in Survey Monkey. The consent form appeared at the start of the survey. Participants were asked to review and indicate whether they wanted to participate in the study. They then completed the survey at some time of their choosing prior to their scheduled phone appointment time. Survey questions asked about health literacy and digital health literacy skills, use of social media and other digital health technologies, and demographics.

We then conducted semi-structured phone interviews at a scheduled time. Phone conversations were conducted on a speakerphone in a private office and recorded. We used a “think aloud” approach with the first five participants. A participant used the program while on the phone with one of the research staff. We asked the participant to talk aloud while they used the system to describe their feedback, including what they liked and disliked about the system. If participants were silent, they were prompted by the facilitator to share their thoughts and opinions (**Table 2**). Once changes were made to the program based on feedback from the first five participants, the second group of five participants completed the program on their own and we spoke with them by phone after they had completed the online program. We asked them for their thoughts about the program using a semi-structured interview. Each participant received a $30 Amazon gift card for participating.

**Table 2:**
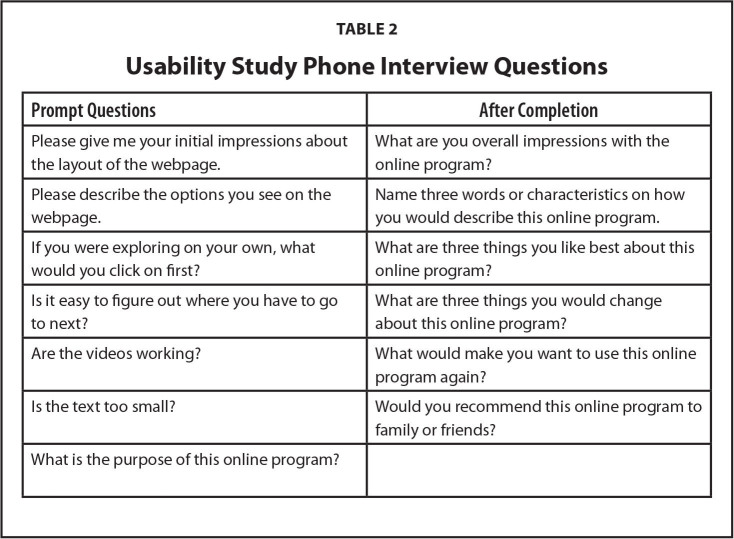
Usability Study Phone Interview Questions

**Prompt Questions**	**After Completion**
Please give me your initial impressions about the layout of the webpage.	What are you overall impressions with the online program?
Please describe the options you see on the webpage.	Name three words or characteristics on how you would describe this online program.
If you were exploring on your own, what would you click on first?	What are three things you like best about this online program?
Is it easy to figure out where you have to go to next?	What are three things you would change about this online program?
Are the videos working?	What would make you want to use this online program again?
Is the text too small?	Would you recommend this online program to family or friends?
What is the purpose of this online program?	

### Pilot Study

Once edits were made to the program based on usability interviews, we began recruitment for the pilot study.

***Recruitment.*** Participants were recruited in a variety of ways. Flyers were posted at various locations around college campuses and the community, such as libraries, doctor's offices, and bulletin boards in stores. We also recruited though word-of-mouth communication, email, and social networking posts, as well as ads on Craigslist. Recruitment materials included a link to the consent form and eligibility questionnaire. Eligibility screener questions (required responses) asked if they were between the ages of 18 and 24, were fluent in English, and had access to the internet. Then participants were provided with the consent form. After reviewing the form, they were asked to indicate willingness to be in the study and provide an ID number per instructions.

Research staff monitored the eligibility survey data. Once reviewed and enrolled, participants were sent a link to complete a pre-test survey on Survey Monkey.

***Measures.*** The pre-test survey asked about demographic information (i.e., age, race and ethnicity, education level, and employment/student status), as well as questions about technology access, use, and social media. We also included the five knowledge questions for each program module to get a pre-test measure. The questions for each module were initially developed by students in public health who had also been involved with the development of the module content. A graduate student and professor who work in educational assessment refined the quiz questions to adhere to best practices. At the end of the survey, participants were directed to another survey to collect an email address so that we could contact them for next steps and to send the $20 Amazon gift card for pre-test survey completion.

eHEALS. The 8-item eHealth Literacy Scale (eHEALS) was administered in both surveys to assess participants' level of eHealth literacy. According to Norman and Skinner (2006), eHEALS “is an 8-item measure of eHealth literacy developed to measure consumer's knowledge, comfort, and perceived skills at finding, evaluating, and applying electronic health information to health problems” (Norman & Skinner, 2006). This scale has proven to be reliable and consistent across multiple studies to capture eHealth literacy levels (alpha = .88, *r* = .51 to .76) (Norman & Skinner, 2006). Scores range from 8 to 40 with lower scores indicating stronger health literacy. Response choices are 1 = *strongly agree*, 2 = *agree*, 3 = *undecided*, 4 = *disagree*, and 5 = *strongly disagree*. **Table 3** shows a list of items.

**Table 3:**
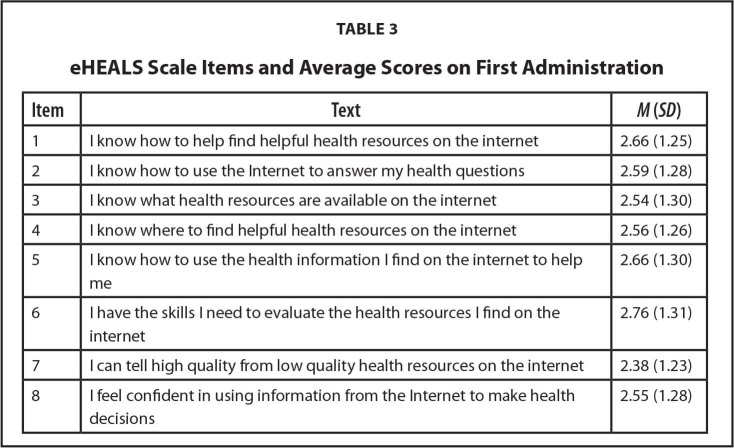
eHEALS Scale Items and Average Scores on First Administration

**Item**	**Text**	***M* (*SD*)**
1	I know how to help find helpful health resources on the internet	2.66 (1.25)
2	I know how to use the Internet to answer my health questions	2.59 (1.28)
3	I know what health resources are available on the internet	2.54 (1.30)
4	I know where to find helpful health resources on the internet	2.56 (1.26)
5	I know how to use the health information I find on the internet to help me	2.66 (1.30)
6	I have the skills I need to evaluate the health resources I find on the internet	2.76 (1.31)
7	I can tell high quality from low quality health resources on the internet	2.38 (1.23)
8	I feel confident in using information from the Internet to make health decisions	2.55 (1.28)

Two weeks after participants completed the pre-test survey, they were sent a link to complete the post-test survey, also in Survey Monkey. This survey included the eHEALS scale and other measures from the pre-test survey to look for changes in knowledge and skills and included usability questions for those who reported using the program. At the end of the survey, participants were taken to another brief survey to enter their information to receive a $50 Amazon gift card. Participants in the control group were sent an email at a later time with information to log into the online program.

***Participants.*** We began the study with 136 participants. Research staff randomly assigned them to either the program or wait-list control group. The program group received information to log into the online program and were instructed to use the program within two weeks. Reminders were sent if they did not log into the program within the first week. Only five did not complete the second survey for a final sample of 131 (wait-list control group *n* = 67; program group *n* = 64). Participants were predominantly female (71.8% female, 28.2% male); 63.4% were White, 16% Black or African American, 13% Asian/Pacific Islander, and 6.1% Native American or Alaskan Native. The remaining 1.5% did not report their race or ethnicity. All but 13.7% of the participants attended some kind of school, with 58% at a four-year college or university and 16% at a community college. Of those who attended school, 67.3% attended school within 30 miles of their permanent residence. More than half (58.8%) belonged to a gym that they had to pay for and 71.8% owned or leased their own car.

***Analysis.*** Repeated measures ANOVA (analysis of variance) was used to determine whether the change in eHEALS and quiz scores, if any, were significantly different for the control and program groups. Both SPSS and R were used to conduct analyses.

***Validity of Participant Responses.*** Unfortunately, participant validity became a concern when we found a wide range of times to complete the survey, some much faster than others. However, after closer inspection, some respondents only completed the multiple-choice quiz questions and not the other survey questions, so it did not make sense to use response time as a rationale for removing participants. Instead, we followed a recommendation by DeSimone and Harms (2018) to not count responses if there was a string of the same response 11 or more times in a row. Based on this rule, we removed 14 participants, resulting in a final sample of 131 participants. This technique made sense given the nature of our surveys, where there would not have been a series of 11 or more questions in a row where the same response would have been reasonable.

## Results

### eHealth Literacy and Health Information Seeking

The mean eHEALS score for all participants was 20.65 (*SD* = 5.60) during the administration in the first survey (alpha = .66); eHEALS scores range from 8 to 40, with lower scores indicating stronger eHealth literacy. **Table 3** shows the average scores per item. The average scores for the two randomly assigned groups, program and wait-list control, were not significantly different, 20.87 compared to 20.42 (*p* = .65), indicating that the random assignment did create equivalent groups with respect to eHealth literacy. A repeated measures analysis of the eHEALS, comparing scores for the program and control groups indicated no significant difference in the improvement in scores over time between the two groups, *F*(1, 129) = 0.003, *p* = .96; however, both groups showed an improvement of 1.0 points on the eHEALS scale. While this small improvement in scores from the first to second administration was significant, there was no practical change in scores (partial eta-squared = .04), meaning the small improvement in scores was the same for the two groups, but not meaningfully significant.

Based on responses during the first survey, most participants reported that they used the internet to look up health information. About 64% of participants agreed or strongly agreed that they look up health information to be more informed (64.1%) or to help manage their own condition (63.4%). Just over half of the participants agreed or strongly agreed that they look up health information to clarify information that was given by a health care professional (54.2%) or to look for alternative or additional treatment options (52.7%). About 45.8% agreed or strongly agreed that they look up health information because they were not provided with enough information during a consultation with a health care professional.

### Pilot Study: Content Validity of Quiz Items

To evaluate the content validity of the six five-item quizzes, we compared participants' responses on specific survey items to the pre-quiz scores. The correlations between the total eHEALS scores and the total pre-quiz scores from four of the six modules were significant and negatively correlated (r = −.24*, −.24*, −.29*, −.14, −.27*, and −.16, respectively, * = *p* < .01), indicating a shared variance between the eHEALS measure and four of the six quizzes. The two quizzes that were not correlated with the eHEALS measure covered social media and wearable health devices, so it is reasonable that there was not a significant relationship between the health literacy measure and those two quizzes, however the content in the other four quizzes is more directly related to health literacy as measured by the eHEALS.

Participants who responded yes to Do you know what a patient portal is? scored significantly higher on the pre-quiz related to patient portals than those who responded no (2.51 compared to 1.76, *p* < .001). The same analysis with the question Have you ever used a patient portal did not result in a significant difference (2.45 to 2.38, *p* = .84), indicating that the quiz questions about patient portals were related to knowledge, even if someone had not actually used one.

Participants who responded that they agreed or strongly agreed with the statement: The health information found on social media is reliable, scored significantly lower than those who disagreed or strongly disagreed, on the quizzes about evaluating health information, with 1.45 correct answers compared to 2.64 (*p* < .001), and social media and health, with 1.64 correct answers to 2.6 (*p* = .007). In addition, participants who responded yes to Have you ever downloaded a health app? scored significantly higher on the quiz related to health apps than those who responded no, 2.42 correct answers to 1.63 (*p* = .004).

### Pilot Study: Pre and Post Quizzes

**Figure 1.**
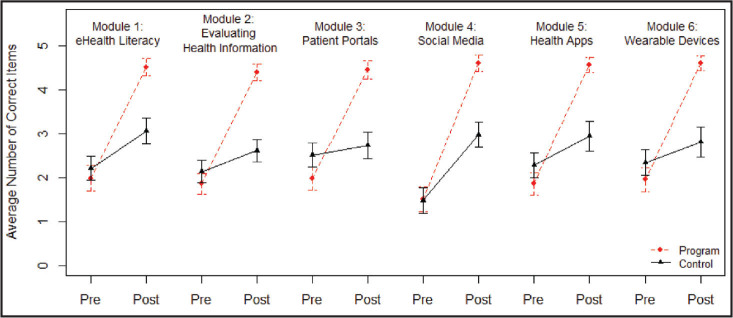
Pre- and post-quiz scores by module for the program and control groups. Note. Error bars represent 95% confidence interval around each mean.

There were no statistically significant differences in pre-test scores when comparing the program and control groups, another indication that we can consider the random assignment successful in creating two equal groups. Using repeated measures ANOVAs, we compared the difference in how much each group improved on the quiz associated with each module (**Table 4**). There were statistically significant differences, with large effect sizes, in the change of pre to post quiz scores by group for each of the six modules, with the program group showing the most improvement. As can be seen in **Figure 1** and **Table 4**, the program group had an average improvement in quiz scores (from pre to post) from 1.6 to 2.3 more correct responses than the control group's improvement across the six modules. The increased improvement for the program over the control group had a large effect size for each of the six quizzes, where a large effect size is a partial eta-squared of .14 or larger.

**Table 4:**
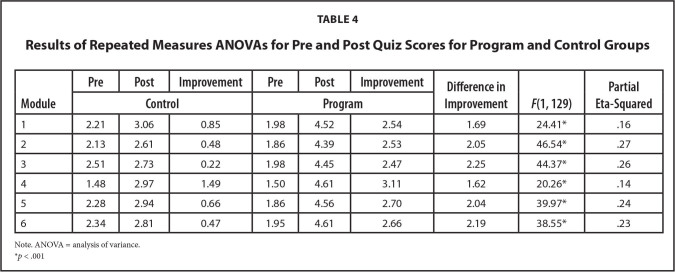
Results of Repeated Measures ANOVAs for Pre and Post Quiz Scores for Program and Control Groups

**Module**	**Pre**	**Post**	**Improvement**	**Pre**	**Post**	**Improvement**	**Difference in Improvement**	***F*(1, 129)**	**Partial Eta-Squared**
	**Control**			**Program**					
1	2.21	3.06	0.85	1.98	4.52	2.54	1.69	24.41*	.16
2	2.13	2.61	0.48	1.86	4.39	2.53	2.05	46.54*	.27
3	2.51	2.73	0.22	1.98	4.45	2.47	2.25	44.37*	.26
4	1.48	2.97	1.49	1.50	4.61	3.11	1.62	20.26*	.14
5	2.28	2.94	0.66	1.86	4.56	2.70	2.04	39.97*	.24
6	2.34	2.81	0.47	1.95	4.61	2.66	2.19	38.55*	.23

Note. ANOVA = analysis of variance.

* *p* < .001

The repeated measures ANOVAs for each quiz were repeated with additional factors of gender, race and ethnicity, and student/employment status. None of these additional factors were significant. For brevity, those results are not presented here.

### Pilot Study: Feedback From Participants About the Program

When asked questions about the program, 67.2% said the information was useful and 59.4% said they learned new things. Concerning the videos, 26.6% said the videos were not necessary while 54.7% agreed they enjoyed watching the videos. About one third (32.8%) thought you could learn just as much if you only watched the videos. Many (42%) thought the quizzes were helpful. Participants felt this program would be appropriate for a range of age groups, and 93.8% said they would recommend it to someone they know. Only seven of the respondents answered the open-ended question: “Do you have any thoughts or comments about the Get Health‘e’ program that we did not ask you about?” Most expressed that they liked the program or enjoyed the study. One provided an important piece of feedback that an explanation of incorrect quiz answers should be provided. The five responses relevant to program content are shown in **Table 5**.

**Table 5:**
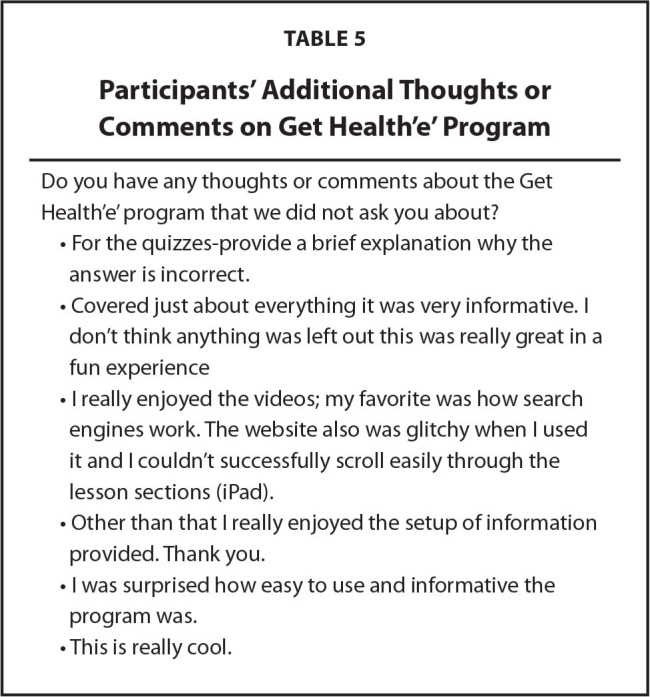
Participants' Additional Thoughts or Comments on Get Health'e' Program

Do you have any thoughts or comments about the Get Health'e' program that we did not ask you about? For the quizzes-provide a brief explanation why the answer is incorrect. Covered just about everything it was very informative. I don't think anything was left out this was really great in a fun experience I really enjoyed the videos; my favorite was how search engines work. The website also was glitchy when I used it and I couldn't successfully scroll easily through the lesson sections (iPad). Other than that I really enjoyed the setup of information provided. Thank you. I was surprised how easy to use and informative the program was. This is really cool.

## Discussion

The Get Health‘e’ tool was successful at increasing knowledge on accessing and evaluating the credibility of health information online as measured by the knowledge questions, although eHealth literacy scores measured by the eHEALS did not change. The tool appealed to young adults, and a large percentage of the intervention group reported that they learned new information, that the tool was useful, and that they would recommend it to someone.

GetHealth‘e’ demonstrates that an online intervention to build digital health literacy skills is feasible and appealing to young adults. This population is connected to digital technology more now than ever before and use the internet frequently for obtaining health information. An online tool to improve digital health literacy is appealing and highly effective at reaching this population. In addition, there was evidence of the content validity of the quiz questions for the GetHealth‘e’ program based on comparison of responses to the survey items and the quizzes. Using technology-based interventions that are accessible on mobile devices is a feasible way to deliver an intervention, even to young adults from vulnerable populations.

Although the eHeals scores did not change, because the questions are not an exact match with content taught, detecting no changes in scores may not be a true measurement of the intervention. While eHEALS is the most commonly used measure of digital health literacy (Faux-Nightingale et al., 2022), there are updated tools such as the Digital Health Literacy Instrument (DHLI) (van der Vaart & Drossaert, 2017), and others (Liu et al., 2021), that are more comprehensive and could be used in future evaluations. eHeals was developed in 2006 before social media and smartphone applications were widely used, so it is focused on basic internet skills and information only. It is also important to note that there are limitations with all measurement tools. For example, a study of the Digital Health Literacy Instrument using cognitive interviewing with adolescents found that there were some places where question wording could be clarified (E. Park & Kwon, 2021). Validated scales can be useful tools for intervention research, but designing specific questions related to program content, as we did in this study, offers another important way of measuring change in knowledge that more directly matches program objectives. In addition, it may be that health literacy and digital health literacy skill measures can have an unexpected relationship with outcomes of interest. People may rate themselves lower once they better understand the complexity of assessing information. As an example, in a study of health literacy and trust in hospitals, results showed that people with the lowest and highest health literacy scores had the lowest trust in public hospitals, but for different reasons (Bertram et al., 2021). More research is needed to understand this relationship.

While digital health literacy is often used to describe skills needed to search for and use information found online, there are other technologies that young adults may need to interact with for health-related purposes. According to Kim and Xie (2017), eHealth refers to “health services and information delivered or enhanced through the internet and related technologies” and can include: electronic communication between patients and providers, electronic medical records, personal health records, health education programs, patient portals and web-based applications (Kim & Xie, 2017). With these technologies, individuals are expected to participate in self-care and self-management of health conditions. For instance, during the COVID-19 pandemic, many places used online technology to schedule COVID-19 tests and check results, and telehealth appointments became widely used. Digital technologies are increasingly used for preventive care (Wong et al., 2020) and managing health conditions such as cancer (Kemp et al., 2021). GetHealth‘e’ is innovative in that it integrates information about some commonly used digital health technologies such as patient portals in addition to developing skills related to online health information seeking.

Digital health literacy has become even more important in light of rapidly developing health situations accompanied by frequent information updates. The COVID-19 pandemic highlighted the need for adequate digital health literacy skills, especially for young adults, including college students (Chen et al., 2023; Hadley et al., 2023; Riiser et al., 2020). In March 2020, many universities and colleges across the country shut down and switched to remote learning because of the pandemic. During this time, young adults were likely obtaining most of their COVID-19 information and prevention recommendations from digital sources. Having adequate digital health literacy levels to obtain and understand recommended public health guidelines became imperative to protect public health and limit the spread. A review found that low health literacy and digital health literacy were contributors to the COVID-19 infodemic (Pian et al., 2021), and a study of college students in the United States found an association of high digital health literacy with intention to get the COVID-19 vaccine (Patil et al., 2021).

Building digital health literacy skills through interventions like Get Health‘e’ could increase the likelihood that young adults understand how to search for and evaluate health information. There is a need for “co-designed educational interventions with adolescents and health providers” (Taba et al., 2022), and this pilot test of Get Health‘e’ provides useful information that can be used to improve upon the content, assessment, and design of future programs. There are many ways to provide such education to youth in the United States, such as incorporating it into health and other courses at the high school level and providing mandatory trainings or making it part of the core curriculum at colleges and universities (Patil et al., 2021).

It is of concern that little research has focused on digital health literacy for populations impacted by health disparities (Choukou et al., 2022). Conducting research on digital health literacy and designing interventions with the potential to help address health inequities is an important area for future work. It is also important to use accessible design principles to ensure that online interventions can be utilized by people of all abilities (Americans with Disabilities Act, 2022).

While this study focused on an intervention designed to improve individual digital health literacy skills, it is equally important for organizations and creators of health information and technologies to consider how to create health literate information and systems. While it is important to build health literacy and digital health literacy skills in individuals, the COVID-19 infodemic highlighted a health literacy crisis that needs to be addressed at organization, community and policy levels. Training can be provided to help strengthen the digital health literacy of health care professionals (World Health Organization, 2023), who then can play a role in helping build digital health literacy for patients (Busse et al., 2022). There are several recommendations for organizations to become more health literate (Agency for Healthcare Research and Quality, n.d.), and resources such as Health Literacy Online (Health Literacy Online, 2016) and the Clear Communication Index (Centers for Disease Control and Prevention, 2023) provide useful tips to help organizations make health information more accessible. In addition, health information has become increasingly politically charged, leading to public distrust in health authorities and public health figures and susceptibility to misinformation. Rectifying this distrust will require improved communication between communities, health experts, and public health officials, and more proactive approaches to health literacy, such as the World Health Organization's competency framework for health authorities and institutions to manage infodemics (Rubinelli et al., 2022).

## Study Limitations

Although participants were randomized into an intervention and control group, the initial sample obtained was a convenience sample and generalizability is limited. While 42% of the participants in the program group reported that they already knew most of the information provided in the program, we did find a statistically significant improvement in quiz scores after the program, suggestion the program was still useful for most participants.

A key limitation was the system used to create the program. The online platform used to disseminate the Get Health‘e’ intervention was not a learning management system (LMS) and is not easily transferrable. LMSs are digital platforms that are used to create and manage learning programs. Transferring the content to a more widely available LMS system would allow Get Health‘e’ to be more easily accessed and shared. Finally, like many online studies, we had to manage participants who tried to participate more than once or completed the survey in suspiciously short time periods. Future studies will need to take precautionary measures to limit these. Utilizing a video consent or requiring .edu email addresses for studies with college students could reduce these challenges.

## Conclusion

Providing digital health literacy skills to young adults is critical given widespread exposure to health messages online and the frequent use of digital technologies to manage health care. With misinformation and disinformation being widely circulated, ensuring young adults have the ability to decipher and evaluate health information is crucial to ensure positive health outcomes. Digital health literacy skills are needed to navigate online health information and empower people to assess it for accuracy and credibility (Dib et al., 2021). An easily accessible and widely available program to build digital health literacy skills is needed. Future research includes developing the content in a more accessible platform and building additional modules to address other aspects of health literacy such as communicating with health care providers and navigating health insurance.
